# An efficient and easy-to-use protocol for induction of haploids in cucumber through parthenogenic embryo development

**DOI:** 10.1186/s40529-024-00436-w

**Published:** 2024-10-08

**Authors:** Pradeepkumara N, Chander Parkash, Reeta Bhatia, Anilabha Das Munshi, Mahesh Rao, Subhashree Subhasmita, Tusar Kanti Behera, Shyam Sundar Dey

**Affiliations:** 1 Division of Vegetable Science, ICAR-Indian Agricultural Research Institute, New Delhi, India; 2 ICAR-Indian Agricultural Research Institute, Regional Station, Katrain, Kullu, Himachal Pradesh India; 3 Division of Floriculture and Landscaping, ICAR-Indian Agricultural Research Institute, New Delhi, India; 4 ICAR-National Institute for Plant Biotechnology, New Delhi, India; 5 ICAR-Indian Institute of Horticulture Research, Bengaluru, India; 6 ICAR- Central Institute of Temperate Horticulture, Regional Station, Dirang, India

**Keywords:** Cytology, Gamma irradiation, Gynoecy, Haploids, Mapping populations, Parthenocarpy

## Abstract

**Background:**

Cucumber (*Cucumis sativus* L.) is a model crop to study cell biology, including the development of haploids and doubled haploids in vegetable crops. In plant breeding, haploid and doubled haploids are valuable tools for developing pure homozygous inbred lines and accelerating genetic progress by reducing the time required for breeding cycles. Besides, the haploids are also valuable in genomic studies. We are reporting the induction of haploids in cucumber involving gynoecious and parthenocarpic genotypes for the first time. This study aimed to assess the efficient induction of haploids through pollination with gamma-irradiated pollen in cucumber. The effect of gamma irradiation dose on pollen viability and germination, fruit setting percentage, seed development, and haploid embryo development in cucumber hybrid genotypes were studied in detail. The goal was to utilize this information to produce haploid plants for genomics and transformation works in this model vegetable crop.

**Results:**

Pollination was done on six cucumber genotypes using varying doses of gamma rays (100, 200, 300, 400, and 500 Gy). Genotypes, doses of irradiation, and embryo developmental stage influenced the successful generation of in-vitro haploid plants. The optimal timeframe for embryo rescue was found to be 25 to 30 days after pollination. Haploid embryos were effectively induced using irradiated pollen at 400 to 500 Gy doses. Parthenogenetic plantlets were analyzed, and their ploidy level was confirmed through stomatal physiology, cytology (mitosis), and flow cytometry methods.

**Conclusion:**

Through parthenogenic embryo development, it is possible to induce a large number of haploids in cucumber. This technique’s power lies in its ability to streamline the breeding process, enhance genetic gain, and produce superior cultivars that contribute to sustainable agriculture and food security.

## Background

The cucumber (*Cucumis sativus* L. 2n = 2x = 14), a member of the cucurbitaceous family, holds significant economic value and is widely utilized as a model species in vegetable crop breeding and functional genomics research (Dey et al. 2022). Cucumber is a highly significant vegetable crop cultivated globally in diverse agro-climatic conditions, including open fields and protected environments. Its origins can be traced back to India, while it also exhibits a secondary center of diversity in China and the Near East. As an outbreeding crop without inbreeding depression, cucumber serves as an excellent model crop for exploring various genetic and molecular pathways due to its unique breeding behaviour and relatively smaller genome size (372 Mbp) (Huang et al. 2009). The classification of different *Cucumis* species into the primary, secondary, and tertiary gene pool is established through evaluations based on cross-compatibility, genetic analysis, phylogenetic studies, and molecular evidence (Dey et al. 2022).

In vitro doubled haploid (DH) plants obtained through androgenesis or gynogenesis techniques offer valuable advantages for both fundamental research and practical applications, such as creating mapping populations, developing inbred lines, and diversifying genetic variability (Segui-Simarro 2021; Ferrie 2011). In hybrid breeding, the required parental lines are traditionally developed through inbreeding. The inbreeding process typically requires 6 to 8 years for cucumber to develop homozygous inbreds. The development of a high-yielding cucumber variety with desirable traits requires hybrid breeding. Homozygous inbred lines are essential as parental lines to create hybrids. These homozygous inbred lines can be generated through the induction of doubled haploids (DHs) using gynogenesis and androgenesis approaches in many vegetable crops (Behera et al. 2022; Dey et al. 2022). Development of haploids using the gynogenesis approach holds greater potential than androgenesis methods, particularly in cucurbitaceous crops. Studies revealed that female haploid cells (present within the embryo sac/ovules) are significantly more responsive to haploid embryo induction than male haploid cells (microspores/pollen) produced within the same flower. In Solanaceous crops like potato, brinjal, tomato, chilli and cruciferous like cauliflower and cabbage, root crops like carrot showed significantly more positive responses for anther culture technique for haploid induction (Bhatia et al. 2018; Mineykina et al. 2021; Romanova et al. 2023; Mulyana et al. 2023; Zhang et al. 2023). Haploid cells are found within ovules, enclosed by nucellar and integument tissue layers, and deep within the ovary. In contrast, male haploid cells are only present within the anther wall. This structural difference makes ovary culture more complex and time-consuming, demanding advanced technical skills.

In recent years, various regions have experienced the emergence of new pathogens that pose significant threats to crop yield. Among these pathogens, cucumber downy mildew disease caused by *Pseudoperonospora cubensis*, has resulted in the complete devastation of entire crops within a short period. Other pathogens like zucchini yellow mosaic virus (ZYVMV) have adversely affected fruit quality and yield, rendering them unsuitable for market. Since resistance to these pathogens is governed by recessive genes, utilising haploids in breeding becomes utmost important for effectively addressing these challenges through transformation, point mutation and targeted mutations.

The main obstacle hindering the utilization of haploids in cucumber breeding program is the lack of an efficient protocol for their large-scale induction. An effective system for producing haploids and doubled haploids can be developed and applied in breeding programs for cucurbitaceous crops. It has the potential to significantly reduce the time needed for hybrid breeding (Sauton 1988a). Induction of haploid plants in various cucurbitaceous crops such as cucumber, watermelon, bottle gourd, pumpkin and winter squash have been reported with limited success using unfertilized ovary/ovule culture and induced parthenogenesis through irradiated pollen techniques (Suprunova and Shmykova 2008; Diao et al. 2009; Li et al. 2013; Moqbeli et al. 2013; Wang et al. 2015; Malik et al. 2011; Koli and Murthy 2013; Zou et al. 2018; Shalaby 2007; Min et al. 2016 and Kurtar et al. 2018). Most of these reports documented very low success and challenges associated with the entire process of haploid production.The doubled haploid technology, achieved through in vitro gynogenesis, has brought significant advantages for breeders working with cucurbits, carrots, and onions. This method allows for the production of gynogenically derived haploid plants in both monocotyledonous species, such as onion (Bohanec and Jakse 1999), leek (Schum et al. 1993), shallot (Cohat 1994) and maize (Bordes et al. 1997) and dicotyledonous species, including marigold (Kurimella et al. 2021), sugarbeet (Ferrant and Bouharmont 1994), sunflower (Gelebart and San 1987), and gerbera (Tosca et al. 1995). However, there have been only a limited number of published results on haploid induction in cucurbitaceous crops (Pradeepkumara 2023). Gynogenesis refers to regenerating embryos from unfertilized female gametophytes, either in vitro or in vivo (Gémes-Juhász et al. 2002). These embryos possess a significant characteristic of having only one set (x) of chromosomes, making them haploid plants (Maraschin et al. 2005). Inducing female gametophytes to form embryos can be challenging to observe sustainably because the embryo sac is embedded within somatic cell tissues, making it difficult to isolate a single embryo sac for culture. However, in vitro gynogenesis holds unique value, particularly for male sterile lines and dioecious plants, where haploids induced through androgenesis may fail, or their induction rates are too low (Bhat and Murthy 2007).

An alternative method for producing haploid plants within a year involves pollinating the female flower with irradiated pollen, leading the egg cells to develop into embryonic cells through parthenogenesis. This process is followed by an embryo rescue technique (Ebrahimzadeh et al. 2018a). Parthenogenesis has recently become the most commonly used technique to obtain haploid plants in cucurbit species (Dong et al. 2016). Numerous research studies have investigated the impact of pollen irradiation on pollen viability, germination, fruit set percentage, and haploid embryo induction in different fruit and vegetable crops. Ionizing radiations, particularly Gamma rays, have been found to inhibit pollen germination only at high doses ranging from 300 to 500 Gy, which is comparable to the doses required to hinder cell enlargement when division is absent. The use of Gamma rays is favoured due to their economic viability and effectiveness compared to other ionizing radiations. This advantage is attributed to the easy availability and strong penetration power of Gamma rays, allowing for widespread application in improving generative cells and vegetative nuclei of various plant species. In the irradiated pollen grains, the migration of pollen chromosomes and physio-biochemical changes have been observed, leading to pollen chromosome aberrations and physio-biochemical changes in irradiated pollens (Zhang 1991; De Witte 2000). When exposed to high irradiation doses, only one male gamete was found per tube, creating the opportunity for fertilization of either the egg cell or the fused polar nuclei. In the former case, the unfertilized component (fused polar nuclei) may develop into a maternal homozygous diploid. On the other hand, in the latter case, the unfertilized egg cell may undergo haploid parthenogenesis, also known as pseudoembryony, where the irradiated pollen serves as a stimulus for the development of the parthenogenic egg cell.

In this study, we focused on pollen irradiation techniques for inducing parthenogenetic haploids in cucumbers. Various factors play a crucial role in determining the appropriate dose of gamma rays for pollen irradiation to achieve non-viability and prevent germination. Among these factors, we specifically examined the impact of donor genotype and irradiation dose on the rates of formation of embryo-like structures (ELS) and plantlet induction. Additionally, we thoroughly evaluated the fruit set percentage, seed quantity (including healthy and chaffy seeds), and the characteristics of regenerated plantlets. These characteristics were compared to understand the effects of the different treatment conditions. Moreover, we implemented further assessments to distinguish true haploids from in-vitro cultured embryonic plantlets. This involved investigating stomatal physiology, cytology, and flow cytometry techniques to ensure accurate identification and verification of haploid samples. Our comprehensive approach aims to enhance the understanding and application of pollen irradiation for parthenogenic haploid induction in cucumber and provide a solid foundation for large-scale induction of haploids and doubled haploids in cucumber.

## Materials and methods

### Plant materials

The experiment was conducted at the Division of Vegetable Science, ICAR-Indian Agricultural Research Institute, New Delhi. The institute is located at 28.63°N latitude and 77.15°E longitude, with an average elevation of 228 m above mean sea level. This geographical location is situated in the Trans-Gangetic agro-climatic zone of India.

In this study, five F_1_ hybrids (GyCl-15 x DC-48, GyCl-15 x DPaC-9-3, DC-83 x DC-48, DC-48 x DPaC-6, Pusa Barkha x GyCl-15) and an elite genotype (DC-48) were selected as seed parents, while DC-43 and Pusa Uday were used as a pollen source. The genotype GyCl-15 was gynoecious, DPaC-9-3 and DPaC-6 were gynoecious parthenocarpic, DC-83 was monoecious, and DC-48 was a natural mutant with an extended shelf-life. The plants of each of the genotypes were raised under a greenhouse during the spring-summer season of 2023. The greenhouse was maintained at temperatures of 25 ± 2 °C during the day and 20 ± 2 °C during the night. To support plant growth, soluble mineral fertilizers were applied to the soil and leaves. Regular applications of fungicides and insecticides were carried out to ensure plant health.

Before opening and anther dehiscence, the male flowers were collected and exposed to varying doses of gamma irradiation (100, 200, 300, 400, and 500 Gy) using a Co^60^ γ-ray source. To prevent unwanted pollination, the female flowers of donor F_1_ hybrids were bagged before the anthesis. The following day, between 7.30 and 9.00 am, pollination was performed using the irradiated pollen (two male flowers for each female flower). After three to five weeks of pollination, the fruits were harvested, and the embryos were cultured in vitro for further analysis.

### Assessment of pollen viability and pollen germination

The male buds of DC-43 and Pusa Uday were collected one day before anthesis in the early morning. After removing the petals, the anthers were exposed to gamma rays at different doses (100, 200, 300, 400, and 500 Gy) on the same day. Cobalt-^60^ was used as the gamma-ray source with an output of 0.2 Gy/s. The irradiated anthers were then incubated at room temperature overnight to facilitate the bursting of pollen sacs. The irradiated flower buds were kept hydrated by placing them in the PCR plates filled with distilled water and placed under appropriate light conditions to ensure proper anthesis. The pollen viability percentage was determined by staining the pollen with 1% TTC (2, 3, 5-Triphenyl tetrazolium chloride) solution. The TTC solution was prepared by mixing 3 g of sucrose, 1 g of TTC, and 10 ml of distilled water, adjusting the pH to 5.7. A drop of the freshly prepared solution was placed on a microscope slide, and the pollen was gently dehisced using a brush before covering it with a coverslip. Pollen grains displaying an orange or bright red color were considered viable, and the viability percentage was calculated using the formula (Vizintin and Bohanec 2004). For the germination, the anthers were gently squeezed on to a cavity slide in a pollen germination media containing 10% sucrose, 100 gL^− 1^ boric acid, 300 gL^− 1^ calcium nitrate, and 0.6% agar. The pH of the semisolid media was maintained at 5.8 (Vizintin and Bohanec 2004). For pollen germination, isolated pollen was incubated in a dark chamber at 25˚C for 60 minutes. After incubation, photos were taken at 400x magnification using a Nikon ECLIPSE 50 I compound microscope. At least 20–50 pollen grains per treatment were examined under the microscope and classified as germinated, not germinated, or burst. Pollen grains were considered germinated if the length of the pollen tube equaled or exceeded the diameter of the pollen grain. Bursting was identified by an irregular mass of cytoplasm, with starch grains protruding from the pollen. The viability percentage was then calculated using the appropriate formula.

### Embryo rescue

Seeds from flowers pollinated with irradiated pollen were collected 30 days after pollination. To avoid contamination, the harvested fruits were surface sterilization with 70% ethanol for 30 s. The seeds were then extracted and subjected to further sterilization with 1.5% sodium hypochlorite for 15 min, followed by three washes with sterile distilled water.

The embryos were mechanically extracted from treated seeds, and cultured on Petri dishes containing MS (Murashige and Skoog; Duchefa, Haarlem, The Netherlands) enriched with 0.5 mgL^− 1^ naphthalene acetic acid (NAA) 5.0 mgL^− 1^ Zeatin, 0.2 mgL^− 1^ GA_3_, 250 mgL^− 1^ PVP, 30 gL^− 1^ Sucrose and 3-4 gL^− 1^ Gelrite, pH 5.7. The seeds were cultured and excised embryos were incubated in a growth chamber at a temperature of 25 ± 2 °C, with a photoperiod of 16 h of light followed by 8 h of darkness. In each petri dish, embryos of 20 seeds were inoculated, and five plates were used for each replication. The experiment was conducted with three replications. After 2 to 3 weeks, the embryos were visually monitored for signs of embryo development. Once embryos were identified in some seeds, these seeds were carefully opened in aseptic conditions. The embryos were then isolated and cultured on a non-supplemented solid medium. Within 10 to 30 days, the cultured seeds/embryos started to germinate. To ensure multiple plantlets, each of the germinated embryos was multiplied by axillary branching technique on non-supplemented solid medium. To promote root induction and further growth and development, these plantlets were transplanted to a half-strength MS medium supplemented with 1.5 mgL^− 1^ IBA.

### Stomatal study

The ploidy levels of plantlets obtained through the induced parthenogenesis approach were assessed indirectly by visualizing the variations in chloroplast numbers in stomatal guard cells. The sampling was carried out on the abaxial (lower) part of the leaf, specifically on the third leaf position. Approximately, 50 stomata were tested for analysis. The experimental procedure involved the following steps: fresh leaves were collected from the top of the plants and discolored using Carnoy solution. The discolored leaves were then immersed in sterile water for 2–5 min. The leaves were then stained with a 1% I-KI (Iodine-Potassium Iodide) solution for 30 s. Finally, the stomatal guard cells were observed under a microscope at a magnification of 400×, and the chloroplast numbers in these cells were counted.

### Ploidy determination using cytology studies

To analyze the ploidy level, actively growing root tips (1.0–1.5 cm) were excised from the in vitro rooted plantlets and stored at 4^0^C for 24 h in ice-cold water. After 24 h pre-treatment, root tips were immersed in 1:3 (glacial acetic acid: ethanol) fixative solution for 12 h. For cytological analysis, 1–2 mm root tips were hydrolyzed in 1 N HCl at 60°C for 12–13 min and then washed in running tap water to remove the residue of HCl and allowed to dry. After drying, roots were kept in Feulgen stain for 30 min in the dark, then crushed with 45% acetic acid, and the number of chromosomes in the metaphase stage was counted to establish the ploidy level of the plants. 4’,6-diamidino-2-phenylindole mounting-media (DAPI, Sigma) was added to label nuclei, and the coverslip was sealed. The cytological status of the root tips was observed under the Leica DM4 P Light microscope at 400 magnification.

### Flow cytometry studies for determination of ploidy

Samples were analyzed for ploidy levels using flow cytometry. Briefly, a small part of tender leaf tissue from reference standard and shoots of unknown ploidy, obtained through induced parthenogenesis were finely chopped with a fine pair of scissors in 1 ml hypotonic propidium iodide lysis buffer (Krishan 1975; buffer with minor modification) having sodium citrate tribasic dehydrate (Sigma-Aldrich, cat. no. S4651), 2 mg/ml RNase A (Sigma-Aldrich, cat.no. P4875), 50 µg/ml PI (Sigma-Aldrich, cat. no. P4170), and 0.3% (v/v) Tween-20 (Sigma-Aldrich, cat. no. P9416). To decrease the effects of cytosolic and phenolic compounds on propidium iodide fluorescence, β-mercaptoethanol (1%) and PVP-40 (1%) were also added to the buffer. After chopping, the suspension was filtered through a 10-µm cell strainer (CellTrics, Sysmex, Cat no. 04-0042-2314) and collected in 1.5 ml Eppendorf tubes. After 15 min incubation, the suspension of isolated nuclei was run on a flow cytometer.

For ploidy analysis, reference control was used as an external standard. Reference control and samples of unknown ploidy were acquired using a flow cytometer (BD Accuri C6, USA) equipped with 552 nm laser and 585/42 Band Pass filter. For each sample around 1000–3000 nuclei were collected and analyzed. Detector settings were set using diploid control and the same settings were used to run the samples. The results were displayed as one-parameter histograms with G0/G1 peak on a linear scale. The fluorescence intensity (median value) of G0/G 1 was recorded for reference control and samples. The CV of G0/G1 peaks were less than 5% for reference controls and samples. The analysis of the data was done using FCS Express Software (DENOVO software, USA) (Krishan 1975). The ploidy level of the samples was determined using the formula: Sample ploidy = reference ploidy × (median value of sample G0/G1 peak)/ (median value of standard G0/G1 peak).

### Statistical analysis

The data analysis for the experiments followed a Completely Randomized Design (CRD) with three replications to assess the effects of the various factors under consideration. ANOVA for statistical analysis was conducted using the SAS (Statistical Analysis System) data analysis software to analyze the variance in the collected data (SAS Institute 1999). The test of significant difference among treatments was calculated using the Turkeys’ Honest Significant Difference (HSD) Test at (*p* ≤ 0.05) using SPSS statistical package.

## Results

### Effect of gamma irradiation on pollen viability and pollen germination

**Fig. 1:**
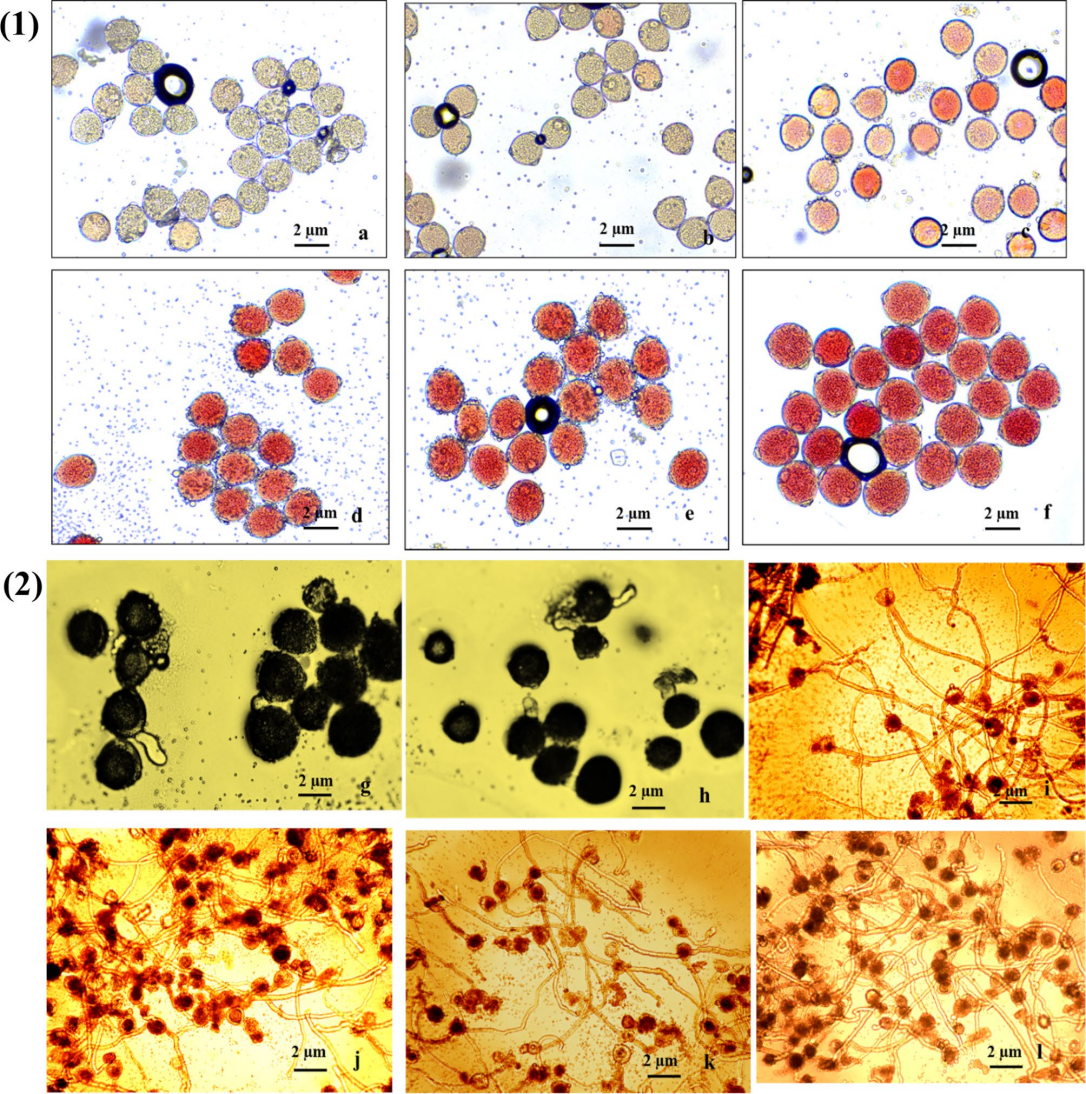
Effect of gamma irradiations on (1) pollen viability and (2) pollen germination (**a, g**)- 500 Gy, (**b, h**)- 400 Gy, (**c, i**)- 300 Gy, (**d, j**) − 200 Gy, (**e, k**) 100 Gy and (**f, l**) Control (0 Gy)

**Fig. 2:**
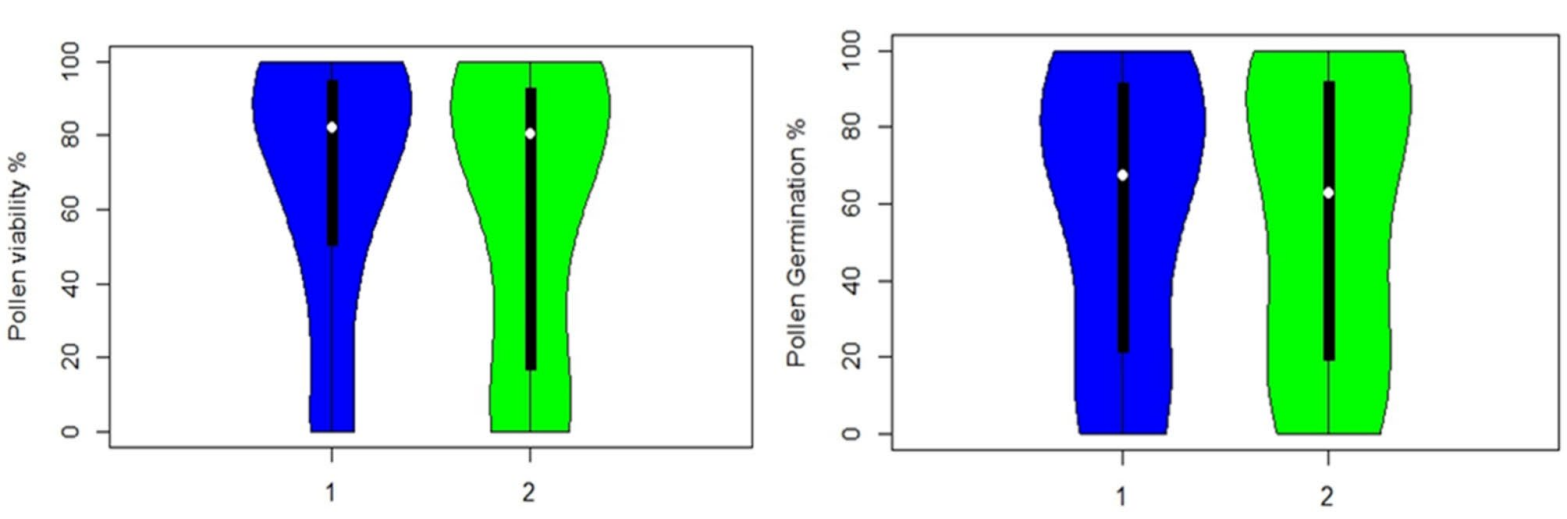
Violin plot: Mean frequency distribution for pollen viability (**a**) and pollen germination (**b**) between two genotypes (1.DC-83 and 2. Pusa Uday)

Our observation on various factors, including irradiation doses, irradiation time, pollen ages, and the response of genotypes to different treatments, were undertaken as per protocols available (Vizintin and Bohanec 2004). The TTC (2,3,5-triphenyl tetrazolium chloride) method was used to assess the viability of cucumber pollen grains under both irradiated and non-irradiated conditions. The investigation revealed that various factors, including irradiation doses, time, stage of the pollen, and the genotype’s response to treatments, pollen viability and germination. As the doses of gamma radiation increased, along with extended irradiation periods and pollen age, a consistent decline in pollen viability was observed compared to non-irradiated pollens. The efficiency of damage caused by gamma irradiation was more pronounced at a dose of 500 Gy compared to doses ranging from 100 Gy to 400 Gy. The lowest pollen viability and pollen germination was recorded when the pollen was irradiated at the doses of 500 Gy (T_5_) (DC-43: 0.00 ± 0.00; Pusa Uday:0.0 ± 0.00) followed by 400 Gy (T_4_) was ( DC-43,49.7 ± 1.52; Pusa Uday, 19.5 ± 16.63 ^c^), Even the lowest radiation dose of 100 Gy caused a significant decline in viability percentage (DC-43, 96.5 ± 2.55 and Pusa Uday, 93.1 ± 1.08) and germination percentage (DC-43, 94.0 ± 4.32 and Pusa Uday, 92.9 ± 2.62^b^) (Table 1**).** Meanwhile, 200 and 300 Gy doses showed less response towards viability and germination percentage than 400 and 500 Gy **(**Fig. 1**).** This implies that higher gamma radiation doses had a more significant impact on pollen viability and pollen germination. Violin plots for comparing the (mean frequency distribution) genotype responses for pollen germination and pollen viability show significantly different responses **(**Fig. 2). Correlation analysis between both the pollen attributes on irradiation dose effect were highly significant (0.97).

**Table 1:** The effect of gamma irradiation dose on pollen viability and pollen germination percentage

Treatment	Pollen viability percentage				Pollen germination percentage			
	Gamma Irradiation Dose (Gy)	DC-43	Pusa Uday	Mean	Gamma Irradiation Dose (Gy)	DC-43	Pusa Uday	Mean
**T**_**0**_	**Control**	100 ± 0.00 ^a^	100.0 ± 0.00 ^a^	100.0	**Control**	100 ± 0.00 ^a^	100.0 ± 0.00 ^a^	100.0
**T**_**1**_	**100 Gy**	96.5 ± 2.55 ^a^	93.1 ± 1.08 ^ab^	94.8	**100 Gy**	94.0 ± 4.32 ^a^	92.9 ± 2.62 ^ab^	93.4
**T**_**2**_	**200 Gy**	86.9 ± 2.60 ^ab^	82.9 ± 4.94 ^ab^	84.9	**200 Gy**	72.2 ± 1.63 ^b^	79.7 ± 8.96 ^b^	75.8
**T**_**3**_	**300 Gy**	80.4 ± 0.90 ^b^	68.7 ± 13.59 ^b^	74.6	**300 Gy**	63.3 ± 1.25 ^c^	48.3 ± 4.99 ^c^	55.8
**T**_**4**_	**400 Gy**	49.7 ± 1.52 ^c^	19.5 ± 16.63 ^c^	34.6	**400 Gy**	21.3 ± 1.25 ^d^	18.3 ± 2.49 ^d^	19.8
**T**_**5**_	**500 Gy**	0.00 ± 0.00 ^d^	0.0 ± 0.00 ^c^	0.0	**500 Gy**	0.00 ± 0.00 ^e^	0.7 ± 0.94 ^e^	0.3
**Mean**		68.9	60.70			58.4	56.6	
**CD (5%)**		3.63	19.85			4.44	9.81	

Values followed by the same letter are not significantly different as determined by **Tukey’s grouping for means of variation** (*p* ≤ 0.05)

### Effect of Gamma irradiated pollen on in-vivo growth parameters and fruit development

While pollen germination and fruit set were more adversely affected by pollen irradiation, fruit growth parameters were also significantly reduced. This effect was relatively irregular in the second season of kharif crop (2023, October), which was marked by a higher weight of fruits than in the first season summer crop (2023, April). The decline in fruit setting (30–100%) (Table 2), fruit length (9–25 cm) (Table 3), and fruit weight (180–450 g) (Table 3) were observed on irradiation. No significant differences were found unless the genotype and environmental interaction strongly support fruit growth and development. The shape of the fruits showed non-marketable (crook neck, bent neck, and circular shape fruits) from the treatment doses like T_4_-400 Gy and T_5_-500 Gy. The chances of less fruit setting percentage were higher with the flower pollinated with higher doses of irradiated pollen.

**Table 2:** The effect of gamma irradiation dose on fruit setting percentage and number of total seeds per fruit

Treatment	Fruit setting percentage (Pusa Uday)										Fruit setting percentage (DC-48)						
	Gamma Irradiation Dose (Gy)		(GyCl-15 x DC-48) X Pusa Uday)	(GyCl-15 x DPaC-9 − 3) x Pusa Uday	(DC-83 x DC-48) X Pusa Uday	(DC-48 x DPaC-6) X Pusa Uday		(Pusa Barkha x GyCl-15) X Pusa Uday		Mean	(GyCl-15 x DC-48) X DC-43)	(GyCl-15 x DPa-C-9 − 3) X DC-43		(DC-83 x DC-48) X DC-43	(DC-48 x DPaC-6) X DC-43	(Pusa Barkha x GyCl-15) X DC-43	Mean
**T**_**0**_	**Control**		100 ± 0.00 ^a^	100.0 ± 0.00 ^a^	100.0 ± 0.00 ^a^	77.78 ± 31.43 ^a^		100.0 ± 0.00 ^a^		95.56	100 ± 0.00 a	100.0 ± 0.00 a		100.0 ± 0.00 a	55.56 ± 31.43 a	100.0 ± 0.00 a	91.11
**T**_**1**_	**100 Gy**		55.56 ± 0.00 ^ab^	55.56 ± 15.72 ^a^	88.89 ± 15.71 ^a^	77.78 ± 31.43 ^a^		55.56 ± 15.71 ^a^		62.22	55.55 ± 31.43 ab	55.56 ± 15.72 a		88.89 ± 15.71 a	77.78 ± 31.43 a	77.78 ± 15.71 a	71.11
**T**_**2**_	**200 Gy**		44.44 ± 15.72 ^ab^	44.44 ± 15.72 ^a^	44.44 ± 15.71 ^a^	55.56 ± 31.43 ^a^		55.56 ± 15.71 ^a^		48.89	44.45 ± 15.73 ab	44.44 ± 15.72 a		44.44 ± 15.71 a	77.78 ± 31.43 a	77.78 ± 31.43 a	57.78
**T**_**3**_	**300 Gy**		66.67 ± 0.00 ^ab^	66.67 ± 27.21 ^a^	77.78 ± 31.42 ^a^	55.56 ± 31.43 ^a^		55.56 ± 31.42 ^a^		64.44	77.78 ± 15.71 ab	55.56 ± 31.43 a		88.89 ± 15.71 a	77.78 ± 31.43 a	44.44 ± 15.71 a	68.89
**T**_**4**_	**400 Gy**		55.56 ± 15.71 ^ab^	55.56 ± 15.71 ^a^	66.67 ± 27.21 ^a^	55.56 ± 15.71 ^a^		77.78 ± 15.71 ^a^		62.22	66.67 ± 27.22 ab	44.44 ± 15.71 a		77.78 ± 31.43 a	44.44 ± 15.71 a	44.44 ± 15.71 a	62.22
**T**_**5**_	**500 Gy**		55.55 ± 31.42 ^b^	77.78 ± 31.43 ^a^	77.78 ± 15.71 ^a^	66.67 ± 0.00 ^a^		77.78 ± 31.42 ^a^		71.11	33.33 ± 0.00 b	55.55 ± 31.43 a		88.89 ± 15.71 a	77.78 ± 15.71 a	66.67 ± 27.22 a	64.44
**Mean**			59.26	66.67	75.93	64.81		77.78			62.96	59.26		81.48	68.52	74.07	
**CD (5%)**			34.61	N/A	N/A	N/A		34.62			42.40	N/A		N/A	N/A	N/A	

**Treatment**			**Number of total seeds per fruit (Pusa Uday)**								**Number of total seeds per fruit (DC-43)**						
		**Gamma Irradiation Dose (Gy)**	**(GCcl-15 x DC-48) X Pusa Uday)**	**(GyCl-15 x DPaC-9 − 3) x Pusa Uday**	**(DC-83 x DC-48) X Pusa Uday**		**(DC-48 x DPaC-6) X Pusa Uday**		**(Pusa Barkha x GyCl-15) X Pusa Uday**	**Mean**	**(GyCl-15 x DC-48) X DC-43)**	**(GyCl-15 x DPaC-9 − 3) X DC-43**	**(DC-83 x DC-48) X DC-43**		**(DC-48 x DPaC-6) X DC-43**	**(Pusa Barkha x GyCl-15) X DC-43**	**Mean**
**T**_**0**_		**Control**	64.00 ± 9.80 ^a^	41.33 ± 15.15 ^a^	47.00 ± 12.25 ^a^		71.33 ± 8.2 ^a^		53.33 ± 19.2 ^a^	55.40	79.67 ± 19.7 ^a^	47 ± 4.2 ^a^	42.00 ± 7.8 ^a^		71.33 ± 8.2 ^a^	53.33 ± 19.2 ^a^	58.80
**T**_**1**_		**100 Gy**	54.00 ± 8.60 ^ab^	47.33 ± 12.26 ^ab^	35.33 ± 17.75 ^ab^		56.67 ± 3.9 ^ab^		29.67 ± 16.7 ^ab^	44.60	66.33 ± 17.4 ^b^	41.00 ± 4.2 ^ab^	32.00 ± 11.2 ^b^		56.67 ± 3.9 ^ab^	29.67 ± 16.7 ^ab^	45.13
**T**_**2**_		**200 Gy**	44.33 ± 6.34 ^abc^	25.67 ± 2.62 ^ab^	35.33 ± 5.79 ^ab^		39.00 ± 6.5 ^bc^		31.33 ± 14.0 ^bc^	35.13	53.00 ± 10.7 ^c^	29.00 ± 5.1 ^bc^	31.00 ± 5.9 ^c^		39.00 ± 6.5 ^bc^	31.33 ± 14.0 ^ab^	36.67
**T**_**3**_		**300 Gy**	30.33 ± 4.64 ^abc^	19.33 ± 3.40 ^ab^	43.00 ± 2.16 ^ab^		26.67 ± 13.10 ^cd^		33.67 ± 6.5 ^cd^	30.60	37.00 ± 7.1 ^d^	24.00 ± 2.4 ^c^	22.00 ± 7.1 ^d^		29.00 ± 10.2 ^cd^	33.67 ± 6.5 ^ab^	29.13
**T**_**4**_		**400 Gy**	32.67 ± 16.74 ^bc^	24.67 ± 7.72 ^ab^	25.00 ± 4.24 ^ab^		20.00 ± 2.16 ^cd^		18.67 ± 4.7 ^cd^	24.20	25.67 ± 5.6 ^e^	18.00 ± 1.6 ^cd^	15.33 ± 6.1 ^e^		18.33 ± 4.5 ^cd^	18.67 ± 4.7 ^ab^	19.20
**T**_**5**_		**500 Gy**	17.67 ± 4.11 ^c^	14.00 ± 2.83 ^b^	13.67 ± 3.86 ^b^		13.33 ± 4.50 ^d^		9.00 ± 1.4 ^d^	13.53	13.67 ± 3.1 ^f^	8.00 ± 3.6 ^d^	11.67 ± 6.0 ^f^		12.33 ± 3.8 ^d^	9.00 ± 1.4 ^b^	10.93
**Mean**			40.50	28.72	33.22		37.83		29.28		45.89	27.94	25.67		37.78	29.28	
**CD (5%)**			26.93	8.18	16.72		14.58		N/A		26.93	8.18	16.72		14.58	N/A	

Values followed by the same letter are not significantly different as determined by **Tukey’s grouping for means of variation** (*p* ≤ 0.05)

**Table 3:** The effect of gamma irradiation dose on fruit length(cm) and fruit weight (g)

Treatment		Fruit length (cm) (Pusa Uday)							Fruit length (cm) (DC-48)						
		Gamma Irradiation Dose (Gy)	(GyCl-15 x DC-48) X Pusa Uday)	(GyCl-15 x DPaC-9 − 3) x Pusa Uday	(DC-83 x DC-48) X Pusa Uday	(DC-48 x DPaC-6) X Pusa Uday	(Pusa Barkha x GyCl-15) X Pusa Uday	Mean	(GyCl-15 x DC-48) X DC-43)	(GyCl-15 x DPOC.9 − 3) X DC-43	(DC-83 x DC-48) X DC-43		(DC-48 x DPaC-6) X DC-43	(Pusa Barkha x GyCl-15) X DC-43	Mean
**T**_**0**_		**Control**	22.00 ± 2.94 ^a^	15.67 ± 3.85 ^a^	15.33 ± 2.49 ^a^	21.67 ± 7.71 ^a^	19.00 ± 5.88 ^a^	18.73	13.67 ± 7.76 ^a^	16.00 ± 0.00 ^a^	22.67 ± 6.65 ^a^		23.33 ± 5.43 ^a^	19.00 ± 5.88 ^a^	18.93
**T**_**1**_		**100 Gy**	10.67 ± 1.24 ^a^	19.67 ± 3.85 ^a^	17.67 ± 4.02 ^a^	19.00 ± 2.16 ^a^	17.00 ± 2.16 ^a^	16.80	9.33 ± 4.71 ^ab^	15.00 ± 4.96 ^a^	16.67 ± 5.55 ^a^		17.67 ± 4.02 ^a^	17.00 ± 2.16 ^a^	15.13
**T**_**2**_		**200 Gy**	20.33 ± 3.68 ^a^	17.67 ± 4.02 ^a^	13.00 ± 2.94 ^a^	17.00 ± 1.41 ^a^	15.67 ± 3.29 ^a^	16.73	14.00 ± 7.78 ^ab^	21.33 ± 8.17 ^a^	9.67 ± 3.09 ^a^		13.00 ± 2.94 ^a^	15.67 ± 3.29 ^a^	14.73
**T**_**3**_		**300 Gy**	15.00 ± 4.24 ^a^	13.00 ± 2.94 ^a^	18.00 ± 5.71 ^a^	13.00 ± 3.55 ^a^	13.33 ± 3.39 ^a^	14.47	13.67 ± 0.47 ^ab^	17.00 ± 2.16 ^a^	26.33 ± 1.88 ^a^		18.00 ± 5.71 ^a^	13.33 ± 3.39 ^a^	17.67
**T**_**4**_		**400 Gy**	15.00 ± 3.55 ^a^	18.00 ± 5.71 ^a^	15.67 ± 3.85 ^a^	17.67 ± 8.01 ^a^	17.33 ± 3.39 ^a^	16.73	12.67 ± 3.39 ^ab^	15.67 ± 2.62 ^a^	19.33 ± 2.05 ^a^		15.67 ± 3.85 ^a^	17.33 ± 3.39 ^a^	16.13
**T**_**5**_		**500 Gy**	16.00 ± 6.37 ^a^	15.67 ± 3.85 ^a^	15.67 ± 2.62 ^a^	14.33 ± 2.86 ^a^	14.00 ± 1.63 ^a^	15.13	14.00 ± 7.87 ^b^	13.33 ± 3.39 ^a^	13.67 ± 2.35 ^a^		16.00 ± 2.82 ^a^	14.00 ± 1.63 ^a^	14.20
**Mean**			16.00	15.67	15.67	14.33	14.00		12.89	16.39	18.06		17.28	16.06	
**CD (5%)**			N/A	N/A	N/A	N/A	N/A		N/A	N/A	8.90		N/A	34.62	

**Fruit weight (gm) (Pusa Uday)**								**Fruit weight (gm) (DC-48))**							
**T**_**0**_	**Control**		401.33 ± 84.62^a^	425.00 ± 137.18 ^a^	201.61 ± 12.57 ^a^	203.31 ± 19.07 ^a^	202.75 ± 8.86 ^a^	286.80	348.00 ± 77.24 ^a^	278.33 ± 70.28 ^a^		331.33 ± 30.35 ^a^	337.33 ± 125.49 ^a^	367.67 ± 30.16 ^a^	332.53
**T**_**1**_	**100 Gy**		288.73 ± 125.45 ^b^	253.33 ± 90.07 ^a^	348.00 ± 15.09 ^a^	337.33 ± 125.49 ^a^	239.33 ± 81.74 ^ab^	293.35	404.67 ± 115.07 ^a^	462.33 ± 51.62 ^ab^		319.00 ± 47.38 ^ab^	321.67 ± 81.42 ^a^	351.67 ± 142.46 ^b^	371.87
**T**_**2**_	**200 Gy**		335.00 ± 154.81 ^c^	337.33 ± 125.49 ^a^	404.67 ± 115.07 ^a^	321.67 ± 81.42 ^a^	315.33 ± 44.23 ^ab^	342.80	401.33 ± 84.62 ^a^	288.33 ± 56.32 ^ab^		470.00 ± 84.27 ^ab^	367.67 ± 30.16 ^a^	452.33 ± 48.58 ^c^	395.93
**T**_**3**_	**300 Gy**		335.00 ± 154.81 ^d^	288.33 ± 56.32 ^a^	315.33 ± 44.23 ^a^	239.33 ± 81.74 ^a^	493.33 ± 60.54 ^b^	334.27	245.67 ± 81.81 ^a^	341.78 ± 139.76 ^ab^		329.33 ± 136.95 ^ab^	434.33 ± 143.04 ^a^	450.67 ± 49.87 ^d^	360.36
**T**_**4**_	**400 Gy**		315.33 ± 44.23 ^e^	316.33 ± 156.18 ^a^	431.00 ± 139.37 ^a^	315.33 ± 44.23 ^a^	306.33 ± 79.00 ^b^	336.87	461.66 ± 43.70 ^a^	201.59 ± 27.70 ^b^		205.86 ± 22.55 ^b^	203.21 ± 22.84 ^a^	188.67 ± 7.61 ^e^	252.19
**T**_**5**_	**500 Gy**		234.33 ± 15.43 ^f^	173.56 ± 12.57 ^a^	206.98 ± 9.44 ^a^	203.24 ± 34.10 ^a^	179.55 ± 13.97 ^b^	199.53	464.34 ± 55.86 ^a^	196.57 ± 5.23 ^b^		184.08 ± 32.56 ^b^	212.57 ± 50.10 ^a^	199.98 ± 24.52 ^f^	251.51
**Mean**			318.29	298.98	317.93	270.04	289.44		387.61	294.82		306.60	312.80	335.16	
**CD (5%)**			N/A	N/A	N/A	N/A	123.36		N/A	158.60		157.29	N/A	146.98	

Values followed by the same letter are not significantly different as determined by **Tukey’s grouping for means of variation** (*p* ≤ 0.05)

### Effect of gamma irradiated pollen on seed development

Fruits obtained after pollination with irradiated pollen contained mainly chaffy seeds and relatively fewer whole seeds. The number of apparently normal seeds decreased sharply, even at the lowest dose of 100 Gy for the two pollen parents (Table 2.). The reduction was even more significant with 300–500 Gy dose-treated pollen, so the number of whole seeds was significantly higher for pollen treated at the lower doses than higher doses. While the weight of fruits remained high; the number of full seeds was reduced by irradiation. Compared to controls, the number of full seeds after irradiation varied considerably among the fruits within the same treatment. The highest total number of seeds in F_1_ hybrids fertilized with the irradiated pollens of DC-43 and Pusa Uday ranged from 80.00 (T_1_:100 Gy) to 5.00 (T_5_: 500 Gy) (Table 4**).** Total number of full and chaffy seeds varied from 40.00 and in T_1_ (100 Gy) to 5.00 in T_5_ (500 Gy) (Table 5**)** while total number of chaffy seeds varied from 20.00 (T_1_:100 Gy) to 2.00 seeds (T_5_:500 Gy) (Table 4**).**

**Table 4:** The effect of gamma irradiation dose on number of full seeds/fruit and chaffy seeds per fruit

Treatment	Gamma Irradiation Dose (Gy)	Number of full seeds per fruit (Pusa Uday)						Number of full seeds per fruit (DC-43)					
		(GyCl-15 x DC-48) X Pusa Uday)	(GyCl-15 x DPOC.9 − 3) x Pusa Uday	(DC-83 x DC-48) X Pusa Uday	(DC-48 x DPaC-6) X Pusa Uday	(Pusa Barkha x Gycl-15) X Pusa Uday	Mean	(GyCl-15 x DC-48) X DC-43)	(GyCl-15 x DPOC.9 − 3) X DC-43	(DC-83 x DC-48) X DC-43	(DC-48 x DPaC-6) X DC-43	(Pusa Barkha x GyCl-15) X DC-43	Mean
**T**_**0**_	**Control**	17.67 ± 13.77 ^a^	14.00 ± 4.32 ^a^	17.33 ± 6.94 ^a^	9.00 ± 7.79 ^a^	9.33 ± 3.68 ^a^	13.47	65.33 ± 52 ^a^	37.00 ± 14.72 ^a^	32.00 ± 11.04 ^a^	62.33 ± 7.76 ^a^	44.00 ± 15.74 ^a^	48.13
**T**_**1**_	**100 Gy**	16.67 ± 7.41 ^ab^	20.33 ± 4.50 ^a^	13.33 ± 8.38 ^a^	18.33 ± 11.47 ^a^	4.67 ± 2.05 ^a^	14.67	46.67 ± 25.38 ^ab^	31.00 ± 13.49 ^ab^	23.67 ± 12.97 ^b^	38.33 ± 11.61 ^a^	25.00 ± 15.89 ^a^	32.93
**T**_**2**_	**200 Gy**	21.00 ± 9.93 ^ab^	5.67 ± 1.25 ^a^	9.00 ± 4.97 ^a^	8.33 ± 2.62 ^a^	7.00 ± 2.16 ^a^	10.2	35.33 ± 15.28 ^ab^	19.00 ± 2.16 ^ab^	26.33 ± 7.31 ^bc^	30.67 ± 3.85 ^a^	24.33 ± 13.47 ^a^	27.13
**T**_**3**_	**300 Gy**	8.67 ± 4.64 ^ab^	4.00 ± 0.82 ^a^	21.33 ± 9.18 ^a^	9.33 ± 7.59 ^a^	5.00 ± 1.63 ^a^	9.67	8.00 ± 4.24 ^b^	13.67 ± 3.85 ^ab^	16.00 ± 7.78 ^bc^	17.33 ± 7.31 ^a^	28.67 ± 8.17 ^a^	16.73
**T**_**4**_	**400 Gy**	14.67 ± 7.32 ^b^	11.67 ± 4.19 ^a^	9.00 ± 3.74 ^a^	4.67 ± 1.25 ^a^	4.33 ± 0.94 ^a^	8.87	11.67 ± 8.9 ^b^	11.67 ± 3.29 ^ab^	10.67 ± 2.35 ^bc^	15.33 ± 2.49 ^a^	14.33 ± 3.77 ^a^	12.73
**T**_**5**_	**500 Gy**	11.00 ± 0.82 ^b^	6.33 ± 3.30 ^a^	6.67 ± 3.09 ^a^	3.33 ± 0.47 ^a^	2.67 ± 0.94 ^a^	6	4.67 ± 2.35 ^b^	6.00 ± 2.94 ^b^	9.33 ± 6.18 ^d^	10.00 ± 4.32 ^a^	6.33 ± 1.24 ^a^	7.27
**Mean**		14.94	10.33	12.78	8.83	5.5		28.61	19.72	19.67	29	23.78	
**CD (5%)**		N/A	7.5	N/A	N/A	N/A		31.44	18.81	N/A	15.27	N/A	

Treatment	Gamma Irradiation Dose (Gy)	Number of chaffy seeds per fruit (Pusa Uday)						Number of chaffy seeds per fruit (DC-43)					
		(GyCl-15 x DC-48) X Pusa Uday)	(GyCl-15 x DPOC.9 − 3) x Pusa Uday	(DC-83 x DC-48) X Pusa Uday	(DC-48 x DPaC-6) X Pusa Uday	(Pusa Barkha x Gycl-15) X Pusa Uday	Mean	(GyCl-15 x DC-48) X DC-43)	(GyCl-15 x DPOC.9 − 3) X DC-43	(DC-83 x DC-48) X DC-43	(DC-48 x DPaC-6) X DC-43	(Pusa Barkha x GyCl-15) X DC-43	Mean
**T**_**0**_	**Control**	17.67 ± 13.77 ^a^	14.00 ± 4.32 ^a^	17.33 ± 6.94 ^a^	9.00 ± 7.79 ^a^	9.33 ± 3.68 ^a^	13.47	14.33 ± 11.09 ^a^	10.67 ± 10.53 ^a^	10.00 ± 3.27 ^a^	9.00 ± 7.79 ^a^	9.00 ± 3.61 ^a^	10.6
**T**_**1**_	**100 Gy**	16.67 ± 7.41 ^ab^	20.33 ± 4.50 ^a^	13.33 ± 8.38 ^a^	18.33 ± 11.47 ^a^	4.67 ± 2.05 ^a^	14.67	19.67 ± 9.84 ^a^	10.00 ± 10.20 ^a^	8.33 ± 4.71 ^a^	18.33 ± 11.47 ^a^	6.61 ± 1.09 ^a^	12.6
**T**_**2**_	**200 Gy**	21.00 ± 9.93 ^ab^	5.67 ± 1.25 ^a^	9.00 ± 4.97 ^a^	8.33 ± 2.62 ^a^	7.00 ± 2.16 ^a^	10.2	17.67 ± 8.34 ^a^	10.00 ± 7.12 ^a^	4.67 ± 2.05 ^a^	8.33 ± 4.71 ^a^	5.65 ± 0.53 ^a^	9.26
**T**_**3**_	**300 Gy**	8.67 ± 4.64 ^ab^	4.00 ± 0.82 ^a^	21.33 ± 9.18 ^a^	9.33 ± 7.59 ^a^	5.00 ± 1.63 ^a^	9.67	29.00 ± 9.63 ^a^	10.33 ± 4.99 ^a^	6.00 ± 1.41 ^a^	11.67 ± 6.24 ^a^	3.70 ± 1.20 ^a^	12.14
**T**_**4**_	**400 Gy**	14.67 ± 7.32 ^b^	11.67 ± 4.19 ^a^	9.00 ± 3.74 ^a^	4.67 ± 1.25 ^a^	4.33 ± 0.94 ^a^	8.87	14.00 ± 7.87^a^	6.33 ± 4.78 ^a^	4.67 ± 3.77 ^a^	3.00 ± 2.45 ^a^	4.12 ± 0.86 ^a^	6.42
**T**_**5**_	**500 Gy**	11.00 ± 0.82 ^b^	6.33 ± 3.30 ^a^	6.67 ± 3.09 ^a^	3.33 ± 0.47 ^a^	2.67 ± 0.94 ^a^	6	9.00 ± 0.82 ^a^	2.00 ± 0.82 ^a^	2.33 ± 0.47 ^a^	2.33 ± 0.94 ^a^	2.00 ± 0.30 ^a^	3.53
**Mean**		14.94	10.33	12.78	8.83	5.5		17.28	8.22	6	8.78	5.5	
**CD (5%)**		N/A	7.5	N/A	N/A	N/A		N/A	N/A	N/A	N/A	N/A	

Values followed by the same letter are not significantly different as determined by **Tukey’s grouping for means of variation** (*p* ≤ 0.05)

**Table 5:** The effect of gamma irradiation dose on stomatal characteristics of cucumber (*Cucumis sativus* L.)

Genotype	Stomatal density (no/mm^2^)		Chloroplast number/stomata		Stomatal diameter (µm)		Stomatal length (µm)	
	Haploid	Normal diploid	Haploid	Normal diploid	Haploid	Normal diploid	Haploid	Normal diploid
**DC-48 X DC-43 (Control)**	-	32	-	8	-	15.27	-	28.39
**(GyCl-15 x DC-48) X DC-43**	22	41	4	8	13.3	15.27	18.07	19.01
**(GyCl-15 x DPaC-9 − 3) X DC-43**	13	39	4	8	13.37	15.90	17.37	27.49
**(DC-83 x DC-48) X DC-43**	18	33	4	8	13.0	14.9	18.88	22.3
**(DC-48 x DPaC-6) X DC-43**	12	31	4	8	12.92	15.26	16	29.11
**(Pusa Barkha x GyCl-15) X DC-43**	12	29	4	8	11.00	13.0	19.03	28.52
**DC-48 X Pusa Uday (Control)**	-	30	-	8	-	15.0	-	29.39
**(Gycl-15 x DC-48) X Pusa Uday**	20	44	4	8	13.00	15.0	17.9	19.3
**(GyCl-15 x DPaC-9 − 3) x Pusa Uday**	15	35	4	8	10.36	14.37	19.3	26.39
**(DC-83 x DC-48) X Pusa Uday**	18	35	4	8	13.28	13.7	18.18	28.22
**(Pusa Barkha x GyCl-15) XPusa Uday**	13	30	4	8	14.62	16.34	19.39	23.1

### Parthenogenetic embryo response under in-vitro culture (embryo rescue)

The doses of irradiation and the genotypes were found to play a crucial role in parthenocarpic embryo development during haploid induction. The effects of 5 different doses of irradiation fertilized were assessed on in-vitro regeneration parameters, like days to embryo regeneration, and in-vitro shoot growth parameters, like shoot length (cm). It was found that the effects of genotypes and treatment doses were significant for days to embryo germination and plant height. The embryo germination was observed early in T_4_ (400 Gy) at 9.81 days when compared with other doses, where embryos showed a response at 11.00 to 12 days T_5_ (500 Gy), T_3_ (300 Gy), T_1_ (100 Gy) T_2_ (200 Gy) **(**Table 6**).** The highest significant variance found from the T_1_ (100 Gy) to T_5_ (500 Gy) treatments for shoot length (cm), all the genotypes of T_1_ to T_3_ 8 to 6 cm was observed, and T_4_ to T_5_ treatment through regenerated embryonic plants ranging from 4 to 5 cm plant length **(**Table 6).

**Table 6:** The effect of gamma irradiation dose on days to in-vitro embryo response and in-vitro grown plant length (cm)

Treatment	Gamma Irradiation Dose (Gy)	Days to in-vitro embryo response (Pusa Uday)						Days to in-vitro embryo response (DC-43)					
		(GyCl-15 x DC-48) X Pusa Uday)	(GyCl-15 x DPaC-9 − 3) x Pusa Uday	(DC-83 x DC-48) X Pusa Uday	(DC-48 x DPaC-6) X Pusa Uday	(Pusa Barkha x GyCl-15) X Pusa Uday	Mean	(GyCl-15 x DC-48) X DC-43)	(GyCl-15 x DPaC-9 − 3) X DC-43	(DC-83 x DC-48) X DC-43	(DC-48 x DPaC-6) X DC-43	(Pusa Barkha x GyCl-15) X DC-43	Mean
**T**_**0**_	**Control**	8.33 ± 0.47 ^a^	13.67 ± 3.30 ^a^	11.67 ± 0.94 ^a^	13.67 ± 0.47 ^a^	7.00 ± 0.82 ^a^	11.87	13.67 ± 0.47 ^a^	13.67 ± 3.30 ^a^	11.67 ± 0.94 ^a^	8.33 ± 0.47 ^a^	7.00 ± 0.82 ^a^	10.87
**T**_**1**_	**100 Gy**	7.67 ± 0.47 ^a^	11.00 ± 0.00 ^ab^	13.00 ± 1.41 ^a^	14.00 ± 0.82 ^a^	7.00 ± 0.00 ^ab^	10.53	14.00 ± 0.82 ^a^	11.00 ± 0.00 ^ab^	13.00 ± 1.41 ^a^	7.67 ± 0.47 ^a^	7.00 ± 0.00 ^ab^	10.53
**T**_**2**_	**200 Gy**	8.00 ± 2.83 ^a^	13.00 ± 1.41 ^ab^	13.00 ± 1.41 ^a^	12.67 ± 1.25 ^a^	7.00 ± 0.82 ^ab^	9.73	12.67 ± 1.25 ^a^	13.00 ± 1.41 ^ab^	13.00 ± 1.41 ^a^	8.00 ± 2.83 ^a^	7.00 ± 0.82 ^ab^	10.73
**T**_**3**_	**300 Gy**	9.67 ± 1.70 ^a^	11.33 ± 1.25 ^ab^	11.67 ± 1.70 ^a^	12.00 ± 1.41 ^a^	8.00 ± 0.82 ^b^	13.53	12.00 ± 1.41 ^a^	11.33 ± 1.25 ^ab^	11.67 ± 1.70 ^a^	9.67 ± 1.70 ^a^	8.00 ± 0.82 ^b^	10.53
**T**_**4**_	**400 Gy**	8.12 ± 0.97 ^a^	9.27 ± 0.09 ^ab^	9.61 ± 0.57 ^a^	14.33 ± 2.62 ^a^	7.70 ± 0.92 ^b^	10.81	14.33 ± 2.62 ^a^	9.27 ± 0.09 ^ab^	9.61 ± 0.57 ^a^	8.12 ± 0.97 ^a^	7.70 ± 0.92 ^b^	9.81
**T**_**5**_	**500 Gy**	9.66 ± 0.51 ^a^	8.17 ± 0.19 ^b^	9.58 ± 0.30 ^a^	14.67 ± 2.62 ^a^	9.98 ± 0.51 ^b^	9.31	14.67 ± 2.62 ^a^	8.17 ± 0.19 ^b^	9.58 ± 0.30 ^a^	9.66 ± 0.51 ^a^	9.98 ± 0.51 ^b^	10.41
**Mean**		12.56	10.07	10.42	13.56	7.78		13.56	11.07	11.42	8.58	7.78	
**CD (5%)**		N/A	3.42	2.57	N/A	1.58		N/A	3.42	2.57	N/A	1.58	

Treatment	Gamma Irradiation Dose (Gy)	In-vitro grown plant length (cm) (Pusa Uday)						In-vitro grown plant length (cm) (DC-43)					
		(GyCl-15 x DC-48) X Pusa Uday)	(GyCl-15 x DPOC.9 − 3) x Pusa Uday	(DC-83 x DC-48) X Pusa Uday	(DC-48 x DPaC-6) X Pusa Uday	(Pusa Barkha x GyCl-15) X Pusa Uday	Mean	(GyCl-15 x DC-48) X DC-43)	(Gycl-15 x DPaC-9 − 3) X DC-43	(DC-83 x DC-48) X DC-43	(DC-48 x DPaC-6) X DC-43	(Pusa Barkha x GyCl-15) X DC-43	Mean
**T**_**0**_	**Control**	5.334 ± 0.16 ^a^	6.39 ± 0.16 ^a^	5.28 ± 0.23 ^a^	5.28 ± 0.23 ^a^	5.28 ± 0.73 ^a^	11.87	5.28 ± 0.19 ^a^	8.05 ± 0.14 ^a^	9.18 ± 0.11 ^a^	7.28 ± 0.12 ^a^	6.50 ± 0.14 ^a^	7.26
**T**_**1**_	**100 Gy**	6.61 ± 0.47 ^a^	6.00 ± 0.00 ^ab^	7.00 ± 1.41 ^a^	6.00 ± 0.82 ^a^	7.00 ± 0.00 ^ab^	10.53	5.28 ± 0 09 ^a^	7.74 ± 0.34 ^a^	8.64 ± 0.49 ^a^	7.27 ± 0.14^a^	7.28 ± 0.12 ^a^	7.24
**T**_**2**_	**200 Gy**	5.00 ± 2.83 ^a^	6.00 ± 1.41 ^ab^	5.00 ± 1.41 ^a^	4.67 ± 1.25 ^a^	7.00 ± 0.82 ^ab^	9.73	5.58 ± 0.59 ^a^	7.90 ± 0.12 ^a^	8.21 ± 0.07 ^a^	7.29 ± 0.18 ^a^	6.92 ± 0.42 ^ab^	7.16
**T**_**3**_	**300 Gy**	5.67 ± 1.70 ^a^	6.33 ± 1.25 ^ab^	5.67 ± 1.70 ^a^	5.00 ± 1.41 ^a^	5.00 ± 0.82 ^b^	13.53	5.30 ± 0.15 ^a^	7.50 ± 0.24 ^a^	8.56 ± 0.37 ^a^	7.31 ± 0.18 ^a^	6.61 ± 0.13 ^ab^	7.06
**T**_**4**_	**400 Gy**	6.12 ± 0.97 ^a^	7.27 ± 0.09 ^ab^	5.61 ± 0.57 ^a^	5.33 ± 2.62 ^a^	5.70 ± 0.92 ^b^	10.81	5.58 ± 0.59 ^a^	5.53 ± 0.35 ^b^	5.77 ± 1.17 ^b^	6.35 ± 0.83 ^b^	4.50 ± 0.56 ^ab^	5.54
**T**_**5**_	**500 Gy**	5.66 ± 0.51 ^a^	5.17 ± 0.19 ^b^	6.58 ± 0.30 ^a^	4.67 ± 2.62 ^a^	6.98 ± 0.51 ^b^	9.31	5.30 ± 0.15 ^a^	4.87 ± 0.48 ^b^	5.78 ± 0.99 ^b^	5.31 ± 0.15 ^b^	5.07 ± 0.69 ^b^	5.23
**Mean**		5.56	6.07	7.42	6.56	0.78		5.39	6.88	7.69	6.8	6.15	
**CD (5%)**		N/A	3.42	2.57	N/A	1.58		N/A	0.75	1.48	0.79	0.9	

Values followed by the same letter are not significantly different as determined by **Tukey’s grouping for means of variation** (*p* ≤ 0.05)

### Stomatal characteristics of the parthenogenetically induced in-vitro regenerated plants

**Fig. 3:**
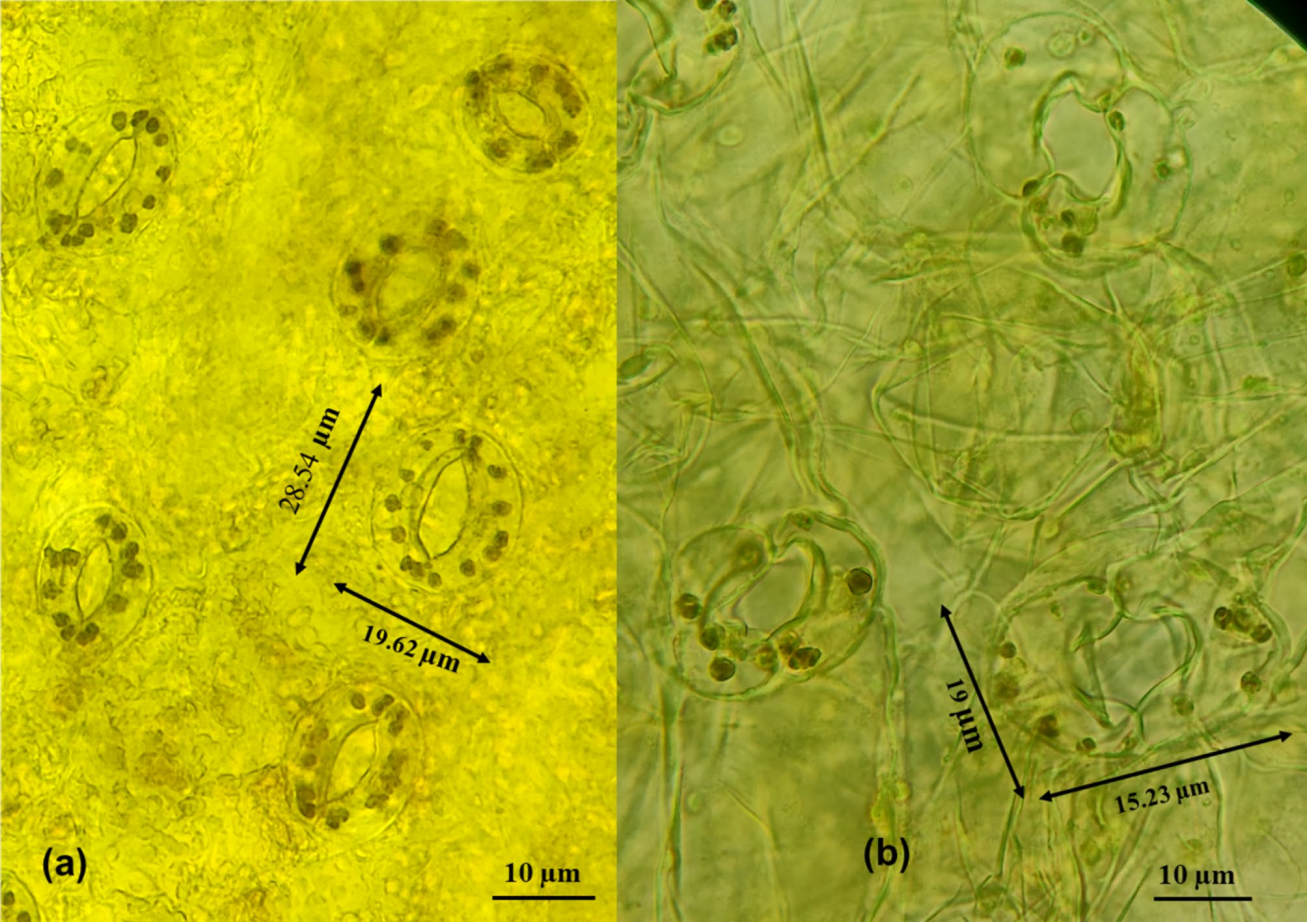
Stomata dimension and chloroplast number in guard cells: Control (**a**) and haploid (**b**)

In the context to in*-*vitro regenerated plants obtained through parthenogenetic haploid induction, distinctions in stomatal length, diameter, and chloroplast count within guard cells were evident between haploid and diploid plants. The average stomatal length in haploid plants (obtained in T_4_ and T_5_) ranged from 16.00 to 19.39 μm, with a diameter spanning from 10.36 to 14.62 μm (Fig. 3**(b)).** Conversely, control plants exhibited higher average stomatal lengths (19.30 to 29.39 μm) and diameters (13.00 to 16.34 μm) (Fig. 3**(a)).** Haploid plants displayed an average chloroplast count of 4 within their guard cells, whereas diploid plants exhibited a count of 8 chloroplasts **(**Table 5; Fig. 3**(b);** Fig. 3**(a))** respectively.

### Ploidy determination by chromosome counting in root tips (mitosis)

**Fig. 4:**
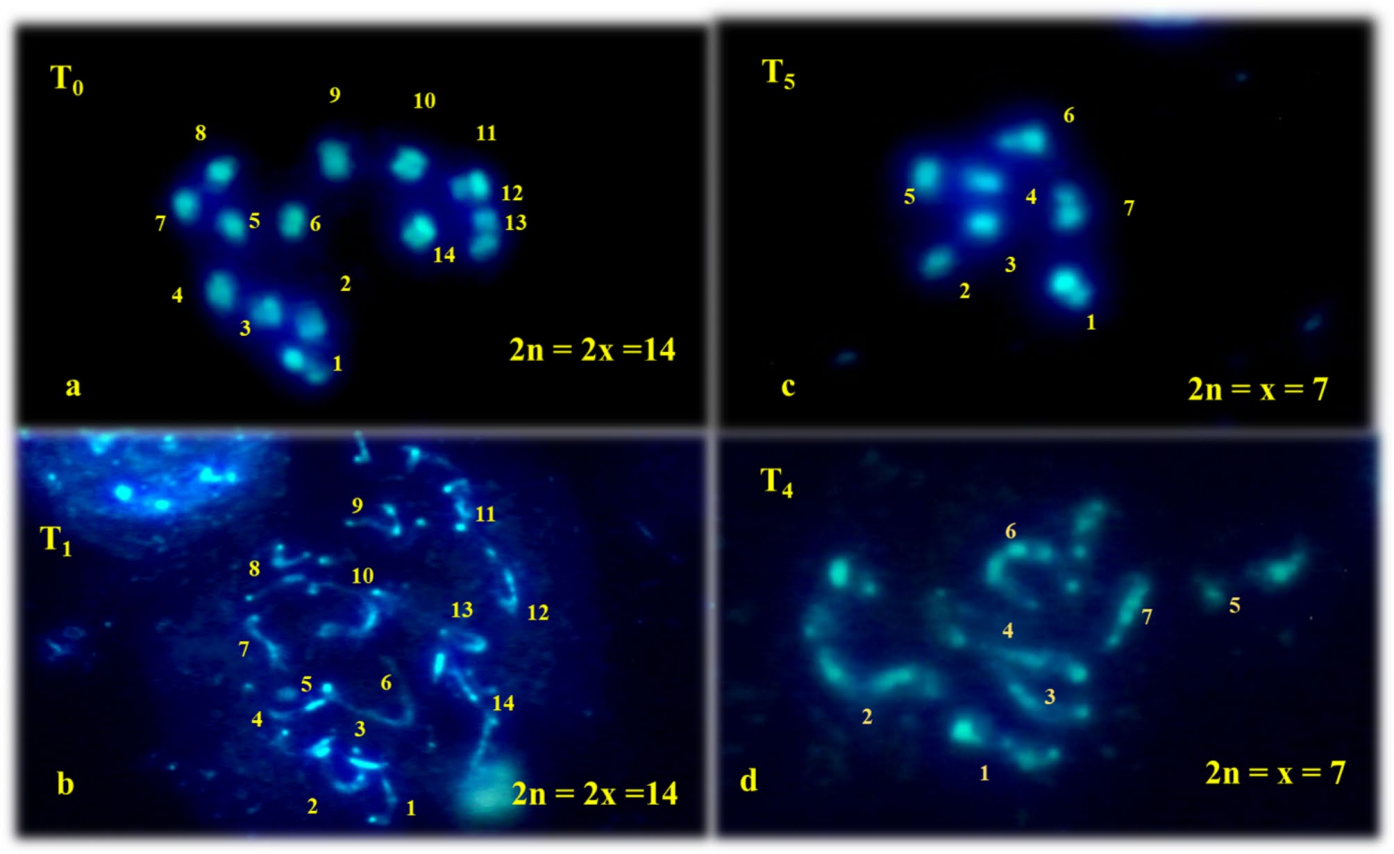
Cytological confirmation of haploid, c and d. (T5 and T4, 2n = x = 7) and diploid, **a** and **b** (Control and T1, 2n = 2x = 14) plants regenerated from irradiated through parthenogenetically induced haploid and control plants (Root tips analysis: Mitosis)

The results of chromosome scoring showed that the ploidy levels of plantlets changed according to embryos developed from different doses of irradiated pollen-treated seeds. Counting the total chromosome number of *in vitro* regenerated plantlets and identifying chromosome morphological characteristics with higher quality metaphase spreads and adjusted the shape of chromosomes. Metaphase plates of diploids (2n = 2x = 14) **(**Fig. 4, a **and b)** and haploids (n = x = 7) **(**Fig. 4, c **and d)** were recorded through cytology. Although the chromosomes were relatively small, the diploid chromosome complement comprised 14 large meta-centric chromosome pairs.

### Ploidy determination by flow cytometry

Ploidy levels of the plants were determined using both direct and indirect methods. The indirect approach involved assessing the morphology of flowers and leaf size, along with evaluating the plants’ growth and fertility, often relying on donor plants as reference points. Although these observable traits provided insight into ploidy levels, confirmation was sought through a more reliable method. Therefore, the direct method involved examining chromosome counts and using flow cytometry analysis on root tips and young leaf samples from in-vitro cultivated plants. In the context of flow cytometric analysis, determining the ploidy level of regenerants resulting from in-vitro embryo rescue relied on comparing their nuclear DNA content to the DNA content of standard donor plants. This allowed for assigning ploidy levels based on the observed nuclear DNA content. Flow cytometry analysis was also employed to identify plants induced through parthenogenesis with varying ploidy levels, including haploids and doubled haploids (DHs), (Bhatia et al. 2017) outlined. DNA median Pi-A value of 53,144.00. In contrast, the diploid normal reference plant shows a DNA median Pi-A value of 1,13,000.0 within peaks.

## Discussion

The results from plants derived from pollination with irradiated pollen subjected to irradiation doses of 100, 200, and 300 Gy suggest that these levels are inadequate for inducing pollen sterility (Froelicher 2007). Lower irradiation doses might only affect a portion of the generative nucleus, preserving its ability to fertilize the egg cell and potentially lead to hybridization (Sestili and Ficcadenti 1996). Haploid individuals were exclusively obtained through irradiation at 550 Gy. Pollen irradiated with 550 Gy through gamma irradiation can germinate on the stigma, traverse the style, and reach the embryo sac. Despite being unable to fertilize the egg cell and polar nuclei, it triggers the development of haploid embryos (Musial 1998). Pollen viability and germination are crucial in supporting fertilization and ovary development, being stable and genetically controlled traits. The effectiveness of irradiation and its impact on pollen are influenced by pollen grain size and shape (Giles and Prakash 1987). Elevated irradiation doses lead to decreased pollen viability and germination rates. Genetic variations among species/genotypes/varieties contribute to the varying resistance levels to irradiation doses. Treating pollen grains with different irradiation doses may reduce their moisture content, limiting their ability to convert carbohydrate reserves. This conversion process is closely tied to the concentration of cytoplasmic water within pollen grains. Such treatment can lead to abnormal meiosis and the formation of irregular gametes, causing notable fluctuations in pollen properties. This, in turn, results in reduced viability and germination potential (Nepi 1993).

Different outcomes were observed at fruit maturity when irradiated pollen was used to pollinate non-parthenocarpic cucumbers. The presence of empty/chaffy seeds became a notable factor contributing to the variation in the overall seed count per fruit comprising both full and empty seeds between the control group and various irradiation doses ranging from 100 to 500 Gy. Interestingly, the number of full seeds determined throughout the entire period of female receptivity displayed no significant disparity between the two seasons. The distinctions in the occurrence of empty seeds predominantly stemmed from the response of the female parent to the irradiated pollen. Our observations revealed that irradiation doses within the 400 to 500 Gy increased the occurrence of parthenogenic haploid fruit set. These fruits contained embryonic seeds with haploid embryos, resulting from pollination and fertilization processes being hindered by the absence or non-functionality of sperm nuclei. In most cases, the pollination of irradiated pollen using higher doses leads to unsuccessful fruit setting, ultimately causing the flowers to wither and drop within 24 h. Moreover, by the clearing method, observation of female flowers pollinated by irradiated pollen at the day of anthesis has shown embryogenesis similar to the control. However, the induced pre-embryos with or without endosperm were abnormal and abortive. This outcome aligns with similar findings reported in prior experiments by researchers such as (Pandey and Phung 1982; James et al. 1985; Sniezko and Visser 1987; Sauton and Dumas de Vaulx 1987). The cucumber crop is chosen as a model for irradiation studies due to its ideal flower size, abundant male flowers, and plentiful pollen availability. Furthermore, the timing of flower anthesis and the duration to achieve full seed maturity are highly convenient for conducting experiments. Based on these cytological findings, we can assert that in cucumber, irradiated pollen triggers stenospermocarpy instead of parthenocarpy. Stenospermocarpy is characterized by fruit development following embryo abortion. However, the precise nature of early embryogenesis, whether it is gynogenetic or involves abnormal zygotic processes, has yet to be definitively established. A small number of gynogenetic embryos develop and subsequently grow into plants after *in vitro* cultivation of mature pseudo seeds. Furthermore, we have noted a seasonal impact (summer and winter) on various growth parameters, including fruit development, seed setting percentage, and gynogenetic response. These observations indicate that the summer season outperforms the winter season due to the crop’s thermosensitive nature.

In this study, a specific combination of 0.5 mgL^-1^ NAA, 0.2 mgL^− 1^ GA_3_, and 5.0 mgL^-1^ Zeatin demonstrated its effectiveness in promoting both the days to embryo response percentage and the length of plant growth within in-vitro conditions. Notably, this combination yielded the highest rate of shoot formation in the plants. The reported use of this particular blend of auxin and cytokinin lays a strong foundation for advancing the development of many haploids and double haploids in cucumber. This approach involves the in-vitro rescue of parthenogenetic seed embryos. In our study, we observed distinct differences in stomatal characteristics between haploid and doubled haploid plants, particularly in size and shape. Notably, counting chloroplasts within the guard cells of stomata emerged as a practical and reliable indirect method for verifying the status of haploid and diploid plants. Comparable outcomes have been documented in various plant species, including *Brassica oleracea, Daucaus carota* and *Cucumis melo* (Abak et al. 1996; Godbole and Murthy 2012), *Capsicum annum* (Abak et al. 1998), *Cucurbita moschata* and *C. maxima *(Kurtar et al. 2018).

**Fig. 5:**
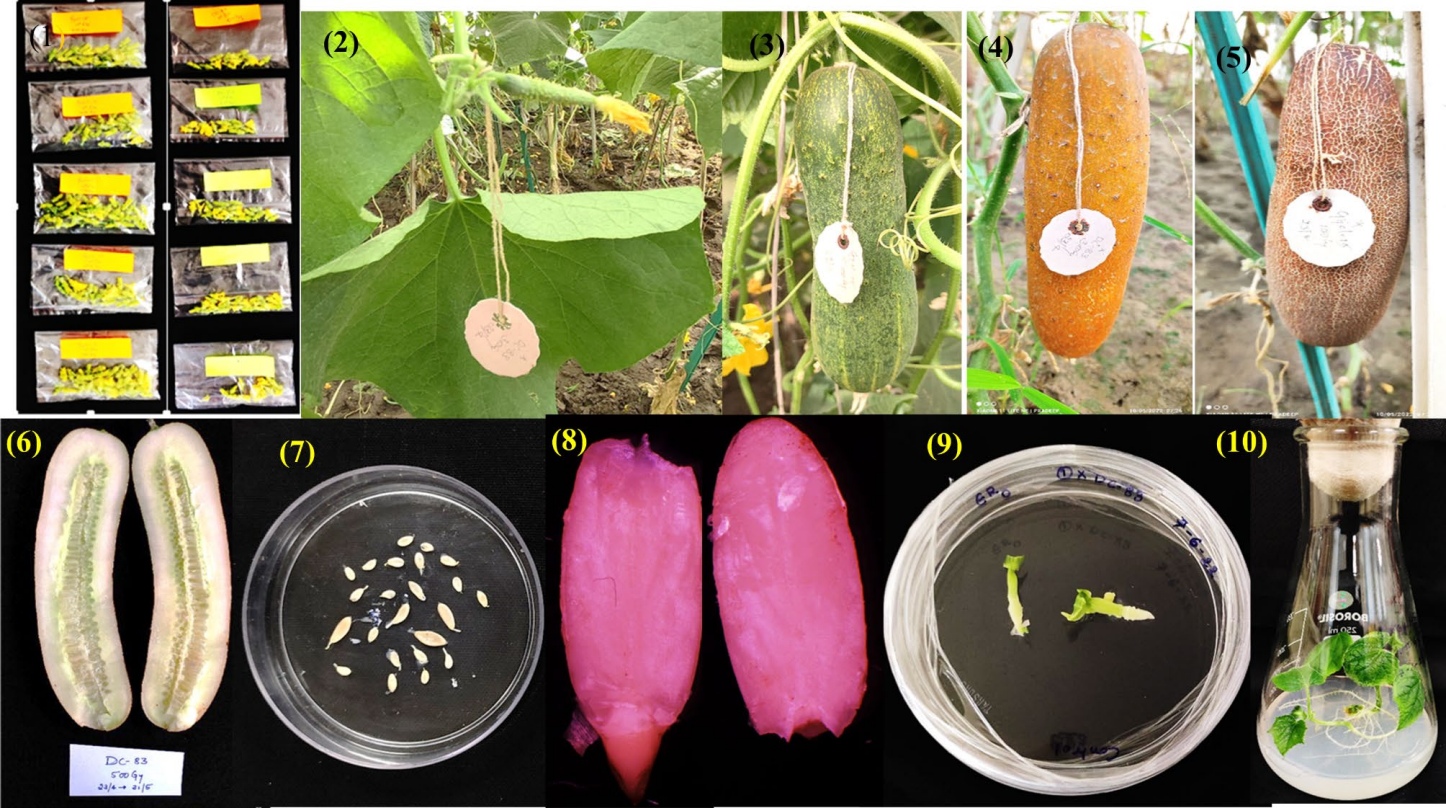
Irradiated pollen through parthenogenetically induced haploids in cucumber. (1) Irradiation of male flower buds at different doses of gamma rays. (2) Pollinated female flower bud. (3) 21 DAP (4) 29 DAP. (5) 37 DAP (6,7) Parthenogenetically induced seed extraction from matured fruit. (8,9) Embryo rescue and in-vitro embryo culture. (10) Plant regeneration from in-vitro cultured seed embryo

In our research, 77 in-vitro regenerated plants underwent root tip cytology (Mitosis). Among these, 23.37% (18 plants) were identified as haploid, while 76.62% (59 plants) were classified as diploid or mixoploid **(**Fig. 5; Table 7**).** Compared to existing literature, the obtained rate of haploidy appears notably satisfactory. In the case of cucumbers and melons, it is plausible that during* in vitro* cultivation, alterations in chromosome count might occur, was studied by flow cytometry studies in many crops (Przyborowski 1994; Ebrahimzadeh et al. 2018). Among the studies available to our knowledge, none of the studies have achieved the haploid induction rate we are reporting in this study (Çetinkaya 2015; Golabadi et al. 2017: Diao et al. 2009). In many crop species, the analysis of stomatal physiology has served as a valuable technique for accurately distinguishing between haploid and diploid plants, as seen in previous studies such as those conducted (Sari et al. 1994) in watermelon and Rode and Dumas de Vaulx (1987) in carrots. The developed protocol will be instrumental in large scale induction of haploids in cucumber for genetic and genomics studies.

**Table 7:** The effect of gamma irradiation dose on ploidy of the parthenogenetically induced regenerated plants of cucumber (*Cucumis sativus* L.)

Irradiation dose	Total number of regenerated plants	Total number of haploid plants	Total number of diploid/mixoploid plants
**Control**	16	0 (n)	16 (2n)
**100 Gy**	11	0 (n)	11 (2n)
**200 Gy**	8	2 (n)	6 (2n)
**300 Gy**	14	2 (n)	12 (2n)
**400 Gy**	13	4 (n)	9 (2n)
**500 Gy**	15	10 (n)	5 (2n)
**Total number of plants**	**77**	**18**	**59**
**Percentage of plants response**	**100%**	**23.37%**	**76.62%**

## Conclusion

We report a significant improvement in the *in vitro*-based development of haploids through an easy-to-use protocol in a model plant, cucumber. This research emphasizes the impact of gamma-irradiated pollen in inducing parthenogenetic haploid plant induction in cucumber (*Cucumis sativus* L.). This method is associated with a notable occurrence of polyembryony and parthenocarpy, facilitating the development of doubled haploids. The combination of pollination using irradiated pollen and the embryo rescue technique allows for the recovery of haploid plants in cucumbers. The findings of the present study indicate successful fruit development triggered by irradiated pollen and the development of haploid embryos as a result of pollination with irradiated pollen. We report a simple regeneration of cucumber pre-embryos from parthenogenetically induced seed embryos rescued at 25–30 days after pollination (DAP), an improvement over previous reports limited to 20–25 DAP. This enhanced efficiency in this simple and easy-to-do embryo rescue procedure is a notable advancement over the earlier studies. Despite recalcitrant, we are reporting a simple protocol for large-scale induction of haploids and doubled haploids in cucumber. In conclusion, the accomplishments outlined in this study will contribute to the ongoing efforts to develop a large number of DH lines in cucumber, thereby facilitating cucumber genetic and genomic studies.

## Data Availability

The data used and analyzed for the current study can be obtained from the corresponding author.

## Abbreviations

DH: Doubled haploid
DAP: Days after pollination
NAA: 1-Naphthylacetic Acid
GA_3_: Gibberellic acid
DNA: Deoxyribonucleic acid
Gy: Gamma irradiation
HSD: Honest Significant Difference
TTC: 2,3,5-triphenyl tetrazolium chloride
